# Unity of Nature and Man: a new vision and conceptual framework for the Post-2020 Global Biodiversity Framework

**DOI:** 10.1093/nsr/nwaa265

**Published:** 2020-10-23

**Authors:** Tianxiao Ma, Yisi Hu, Meng Wang, Lijun Yu, Fuwen Wei

**Affiliations:** CAS Key Laboratory of Animal Ecology and Conservation Biology, Institute of Zoology, Chinese Academy of Sciences, China; CAS Key Laboratory of Animal Ecology and Conservation Biology, Institute of Zoology, Chinese Academy of Sciences, China; University of Chinese Academy of Sciences, China; CAS Key Laboratory of Animal Ecology and Conservation Biology, Institute of Zoology, Chinese Academy of Sciences, China; University of Chinese Academy of Sciences, China; CAS Key Laboratory of Animal Ecology and Conservation Biology, Institute of Zoology, Chinese Academy of Sciences, China; University of Chinese Academy of Sciences, China; CAS Key Laboratory of Animal Ecology and Conservation Biology, Institute of Zoology, Chinese Academy of Sciences, China; University of Chinese Academy of Sciences, China; Center for Excellence in Animal Evolution and Genetics, Chinese Academy of Sciences, China; Center for Evolution and Conservation Biology, Southern Marine Science and Engineering Guangdong Laboratory (Guangzhou), China

People live in nature. However, substantial evidence confirms that, under the pressure of anthropogenic alteration, nature is being fragmented, imperiled and becoming less able to provide essential services [1]. Biodiversity loss is the most significant signal of this depletion, and could profoundly impact the future of human beings and the rest of life on Earth [2].

Against this background, Parties of Convention on Biological Diversity (CBD) agreed a 2011–2020 Strategic Plan and 20 Aichi Targets to halt continuing biodiversity loss. However, according to the latest global assessment report released by the Intergovernmental Science-Policy Platform on Biodiversity and Ecosystem Services (IPBES) in 2019, biodiversity is still declining globally at rates unprecedented in human history [3]. It is clear that the majority of the Aichi Targets will not be met by the 2020 deadline [4]. Even with a careful strategic plan developed and implemented under the authoritative CBD context, biodiversity and ecosystem services critical for humanity are declining and degrading fast. This has prompted reflections on the current strategic plan and the UN framework in general [3,4].

The 15th Conference of Parties (COP15) of CBD will be held in China in 2021, during which the new strategic plan for the next decade of biodiversity conservation will be drafted. Various suggestions have been proposed by scientists and different stakeholders for contributing to this much-anticipated strategic plan. They are focused on meeting the 2050 Vision of ‘Living in Harmony with Nature’. Mace *et al.* [5]argued that targets should be developed in a well-defined, ambitious and measurable way to support the next CBD vision, and that three indicators are required to measure the progress in biodiversity recovery. Paired with the UN’s Paris Climate Agreement, ‘A global deal for nature’ ambitiously targets 30% of Earth to be formally protected and an additional 20% designated as climate stabilization areas by 2030, to preserve biodiversity and keep global warming below 1.5°C [6]. Locke *et al.* [7] proposed an enabling framework of three global conditions for biodiversity conservation and sustainable use that could support both approaches and achieve the 2050 Vision. These technical suggestions provide important ideas for development of the Post-2020 Global Biodiversity Framework (post-2020 GBF). However, they do not question the vision or the basic conceptual framework of the current Strategic Plan of the CBD.

We argue that the current 2050 Vision will not be sufficient to drive transformative change. Further, we argue that human development cannot be divorced from biodiversity conservation and utilization. The basic understanding of the appropriate balance in the relationship between nature and humans is actually the crucial point. It is the starting point that lays the very foundation of any ambitious and effective strategic plan for biodiversity conservation.

With the human-centric perspective dominating for the past two centuries, nature and human are seen as two separate entities [8]. Nature is treated as being ‘outside’ of humans, and thus humans treat nature as an object to fear, conquer, pillage and rule. In this context, nature and man are regarded as opposing entities with contradictory demands. It is true that the two have different needs: nature's demands are to maintain its components, ecological processes and evolutionary potential, while man's demands are to sustain a growing population and improve quality of life relying on resources and services provided by nature. But the assumption underlying this separation of humanity and nature is that nature is a limitless storehouse for humans to enrich themselves as much as their creativity allows. Dominant anthropocentrism and dramatic technological development free humans to exploit nature, which has already exceeded a safe and just operating space for humanity [9,10]. Nature's demands have been neglected in this process, which in turn undermined man's basic needs, including demands under the Sustainable Development Goals for clean air and water, via interactions across the coupled human-natural system [11]. In light of the great damage this thinking has caused to biodiversity and ecosystems, we can no longer assume that nature is an infinite resource to exploit. Nature and its ability to provide services keep being damaged, which we now know threatens the future development of humanity [3]. Consequently, a rethink of the relationship between nature and man, and also their demands, is essential for ensuring the appropriate course of biological diversity conservation and also humanity development for the coming decades.

## ‘LIVING IN HARMONY WITH NATURE’ *VS.* ‘UNITY OF NATURE AND MAN’

The modern nature-human dichotomous perspective emphasizes the material substance of nature and its instrumental value relative to the contribution to humans [8]. However, within the context of some other knowledge systems, nature has its intrinsic value because of the existence of its components and also the broader aspect of concepts it covers, such as the cultural elements of ancestors, shared history and deities [12]. This intrinsic value is not necessarily related to the materials and services nature provides for man but, in turn, man is included as component just as other animals [13]. One extreme example is the traditional Chinese Taoism, in which nature is represented by Tien (Heaven and Earth), which is composed of human and non-human nature and even the ultimate rule of this universe. All these perspectives have a common ground – nature and man are as one.

Sharing this perspective, Taoism describes the relationship between nature and man as ‘Heaven and earth were born at the same time I was, and the ten-thousand things are one with me’ (天地与我并生, 而万物与我为一) [14], which can be concisely summarized as a vision of ‘Unity of Nature and Man’ (‘天人合一’) (UNM). The Chinese sages’ UNM vision embraces inherent respect for nature and advocates that humanity development should conform to the rule of nature with a holistic view. Against the background of the current perspective of separation of nature and man and the resulting ecological crisis, this inspires us to recognize ourselves as, and behave as, a member of nature following the principle of UNM.

The current 2050 Vision uses the words ‘Living in Harmony with Nature’, the meaning of which in its original language is society in symbiosis with nature, both with mutual benefit and necessarily detrimental aspects for one of the parties [12]. However, Living in Harmony with Nature (LHN) as used by the CBD has four attributes which narrow its meaning. The Vision says: ‘By 2050, (b) biodiversity is valued, conserved, restored and wisely used, (c) maintaining ecosystem services, (d) sustaining a healthy planet and (e) delivering benefits essential for all people’.

This version of the meaning of LHN loses its original Taoist flavor. This can be seen in the words that imply nature's value to humans is necessary: nature is to be ‘valued, conserved, restored and wisely used’ as an object, not treated as an equal. There is no recognition that it has its own needs and its own evolutionary direction that must be respected in a relationship of mutuality as opposed to one of exploitation. While realizing this vision would certainly be an improvement over current conditions, it still does not create the indispensable right relationship of UNM on which future sustainability depends. In contrast, a vision based on the Taoist idea of ‘Unity of Nature and Man’ could achieve this.

## 2050 VISION: UNITY OF NATURE AND MAN (UNM)

As UNM implies, nature's intrinsic value is the existence of its components and broad non-material concepts covered. It advocates that man, as one component of nature, must arrange his activities following the rule that nature contains, and without damaging the sustainability of other components when meeting his own needs. For example, in classical Chinese philosophy, UNM proposed ‘树木以时伐焉 (Trees are logged by time), 禽兽以时杀焉 (Birds and beasts are hunted by time)’, telling people to use natural resources conforming to the life cycle of creatures to guarantee the sustainability of both provider and beneficiary. However, along with humanity's overexploitation of nature to accommodate increasing demands for material goods, the modern human-nature relationship has clearly deviated from the ideal state of UNM and led to the current environmental crisis. To halt the continuing decline of nature and revert to UNM, humanity development must be aligned with nature's limits and demands by respecting its existence, conforming to its rule and conserving its sustainability. UNM considers the demands of both nature and man at the same time by adopting a sustainable approach. In this way, nature and man are no longer two opposed individuals, nor are their demands, but are in complete harmony, blend and finally become one.

On 5 September 2019, the theme of CBD COP15 was finalized and announced as ‘Ecological Civilization - Building a Shared Future for All Life on Earth’ [15]. This theme originates from the Chinese vision of ecological civilization. Ecological civilization is an eco-innovation rooted in traditional wisdom of UNM to harmonize the apparent contradiction between economic development and environmental protection [16]. COP15 is considered to be a ‘unique and historical opportunity’ to reconcile the relationship between humanity and nature [15]. UNM, as the real connotation of COP15 theme, could enable this renewed understanding, promote the establishment of ‘a global society in which economic, social, cultural and environmental concerns are addressed in a truly holistic way’ [15] and foster a sustainable future shared by all life on Earth.

To summarize the above implications of UNM, we propose a more holistic 2050 Vision of *‘Unity of Nature and Man whereby all of Nature is respected, its rule is conformed to, and its components are adequately protected and Humanity meets its own unique needs through sustainable production and sustainable consumption on land and ocean, therefore the sustainability of both nature and man are guaranteed and united.’* This UNM Vision can direct and motivate more comprehensive and effective actions for conserving biodiversity and meeting human's demands at the same time to ensure the effective outcome of the CBD’s three main objectives.

**Figure 1.**
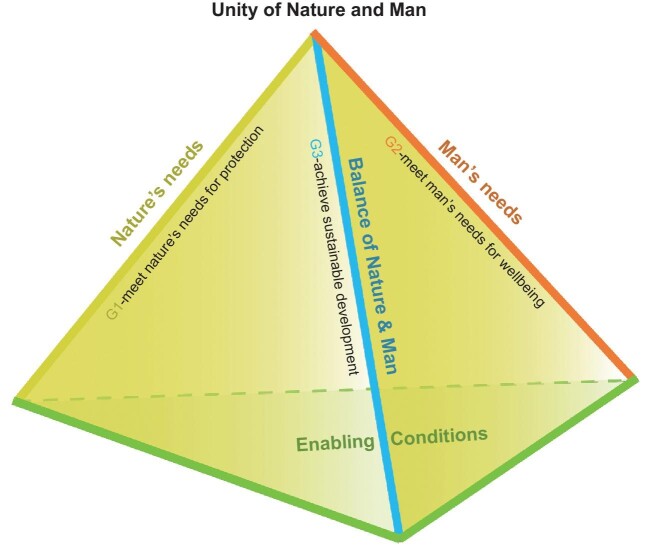
Tetrahedron structure of the new conceptual framework. The three upper skeletons represent the main strategic goals of the framework, focusing on ‘Nature's needs’, ‘Man's needs’ and ‘Balance of Nature and Man’, respectively The base plain represents the enabling conditions (e.g. mainstreaming, capacity building and resource mobilization) for assuring effective implementation of the strategic plan.

Using the Chinese Taoist philosophy, we propose a new conceptual framework to illustrate the vision of ‘Unity of Nature and Man’ and relate it to implementation. A tetrahedron framework shows the three main skeletons, represented by ‘Nature's needs’, ‘Man's needs’ and ‘Balance of Nature and Man’, and their relationships for achieving UNM (Fig. 1).

The base plain in the bottom represents the enabling conditions (e.g. mainstreaming, capacity building and resource mobilization) acting as the footstone of the whole framework. Each of the three skeletons has a clear goal, which combined with the others, will lead towards UNM. The three are (1) meet nature's needs for protection, (2) meet humans’ needs for wellbeing, and (3) achieve sustainable development that leads to a new kind of prosperity which respects nature's needs, and unites and balances the needs of nature and human. These goals also correspond to the three main objectives of CBD including biodiversity conservation, equitable sharing of benefit (wellbeing) and sustainable use of biodiversity. When all three strategic goals are supported by another goal of adequate enabling conditions, we can achieve UNM.

The key point of this is that true integration of nature and man (UNM) means integrating human development goals and biodiversity goals as equal and mutually reinforcing. Both have their own needs which must be met, and where they meet they must be co-equal and inextricably intertwined.

## KEYS FOR THE PATHWAY TOWARDS UNM VISION

While the Unity of Nature and Man provides an alternative conceptual framework to rethink and deal with our relationship to nature, further developments are needed to enable its effective implementation. Here we propose four dimensions that should be examined to enable the changes that can balance the needs of nature and man moving forwards to achieve the strategic goals of CBD and 2050 UNM Vision.

### Transformation of value systems underpins the departure from business-as-usual

Based on the above discussion, the long-term misinterpretation of our relationship with nature and the resulting interferences are responsible for today's environmental crisis. Previous failures evidenced that business-as-usual cannot slow the rate of biodiversity loss, let alone put it on a path to recovery. Departure from business-as-usual will not happen naturally, especially with the opposition from those with vested interests [3,4], but can be underpinned and fostered by alternative value systems of whole societies. Recognition and respect of nature's intrinsic value as well as positioning man as part of nature will motivate people to move to address the problem and seek the ideal state of Unity of Nature and Man. The shift of value systems will enhance individuals’ and societies’ internal connection with nature [17], and drive policy, technology and humanity development on the track towards sustainability. The achievement of global biodiversity targets relies on collective global efforts. Although UNM originates from classical Chinese philosophy, its core is shared by different cultures and perspectives worldwide, e.g. the ancient ‘Mother Earth’, ‘Gaia’ in South America, ‘Brahman’ in India and modern environmental ethics. In this light, UNM is promising to align efforts of different parties and communities to a unified vision. This nature also allows for UNM and its principles to be translated easily into language that facilitates parties and communities developing specific solutions for balancing local developments with nature and sharing useful implementation experiences.

### Holistic view and systems thinking promotes required knowledge and nexus approaches

Interactions between nature and humanity, including between ecosystems and human wellbeing, are complex. To address the current environmental crisis, including biodiversity loss, requires a deepening understand of this complexity, which can be informed by UNM philosophy. Knowledge of coupled human-nature or socio-ecological systems, including that of indigenous and local communities [12], should be accumulated and should evolve through enhanced interdisciplinary research and adoption of a more holistic view of UNM that regards nature and human as an organic whole. As biodiversity is a multifaceted issue intertwined with human development, nexus (integrated) and system-oriented approaches are needed to simultaneously achieve goals of biodiversity conservation, related human wellbeing improvement, and to seek a balance in the UNM framework. Systems thinking focusing on dynamic relationships of these three dimensions, including their elements, is necessary to identify effective solutions to address complex challenges of biodiversity loss and sustainable development. Some good practices are emerging. For instance, the Nature-based Solutions aims to provide human wellbeing and biodiversity benefits simultaneously with support of nature [18], and the policy innovation of Ecological Conservation Redline, under a broader institutional framework of Ecological Civilization in China, is designed to guarantee both the national ecological security and essential ecosystem services [19–21]. These concepts and practices offer references and inspiration for other countries to develop integrated solutions to sustain biodiversity benefits and human wellbeing.

### Transformative changes to tackle indirect drivers of biodiversity loss and concrete commitments of Parties

Based on the Global Assessment Report on Biodiversity and Ecosystem Services issued by IPBES, three goals of CBD and sustainable future cannot be met along current trajectories, and are only possible through transformative changes across economic, social, political and technological factors [3,4]. According to Donella Meadows, there are ‘leverage points’ where small shifts can lead to fundamental changes in the system towards sustainability [22]. Although specific leverage points vary across contexts, a shift of value systems, as well as visions of a good life, are agreed to be deep leverage points for enabling transformative changes from current trends to more sustainable ones [4,17,23]. In this light, the transformation of how we value nature and the way we deal with the relationship to nature proposed by the UNM makes it fundamental momentum and also enhancement of other leverage points for societal changes. Additionally, three goals in the UNM conceptual framework, namely meet nature's needs, meet human’s needs, and their balance through sustainable development, reflect the complex interconnection between biodiversity issues and humanity development. Endeavors to reach these goals will promote knowledge accumulation of social-ecological systems, including externalities and telecouplings, while the UNM philosophy's holistic view can inform and foster integrated solutions for the nexus of relevant goals featured by complex interactions and multi-sector involvement, all of which can underpin desirable transformative changes in different aspects. On the other hand, the realization of UNM Vision and its potential contributions to CBD’s three strategic goals and the Sustainable Development Goals rely on concrete commitments from Parties. The shared aspiration to build a sustainable future for both humanity and nature will form the first step of global communities towards the UNM Vision. Mainstreaming humans’ dependence on nature and the necessity to respect the intrinsic value of nature and needs will help to generate a sense of responsibility to act for nature and biodiversity across sectors in different scales. Parties should take the form of commitments to achieve transformative changes in current unsustainable production and consumption patterns to reduce indirect drivers for biodiversity loss, and in governance approaches to handle nexus challenges on simultaneously meeting the needs of nature and man in the long term. Last but not least, a transformative resource mobilization strategy is essential for sufficient and effective financial support for the ambitious UNM Vision, especially against the background of post-pandemic recovery of the world's economy.

**Figure 2.**
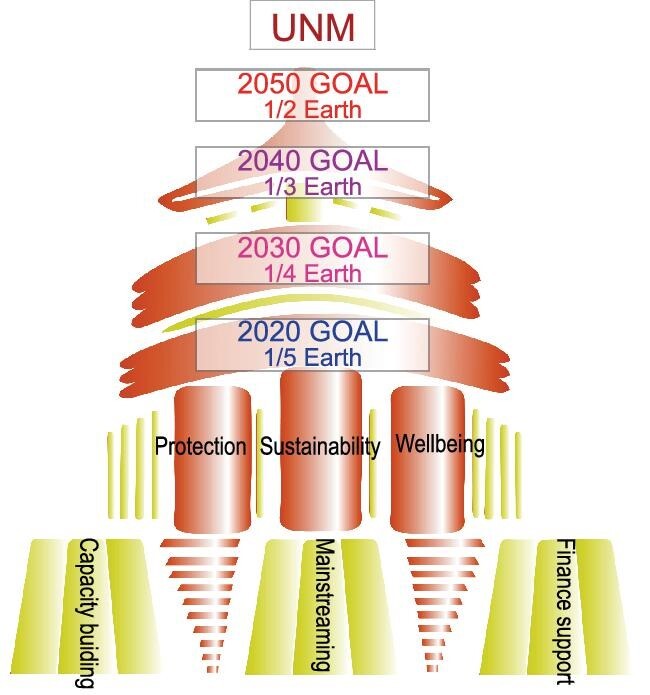
A Chinese temple showing the step path towards the UNM Vision. Standing on the base plain of ‘enabling conditions’, three strong pillars symbolize the three main strategic goals of this new framework. These support upgrading of the stage goals of 2020, 2030, 2040 and 2050. By achieving these goals, we can finally reach the vision of UNM, ‘Unity of Nature and Man’.

### A phased approach and milestones on area-based target to reach the UNM Vision

‘Unity of Nature and Man’ aiming to tackle the long-term imbalance of meeting the needs of nature and man is an ambitious vision. It can be reached only by achieving stage goals one by one following periodic strategic plans with collective global efforts. Milestone(s) or overarching goal(s) can motivate willingness of stakeholders to develop ambitious but realistic plans on biodiversity conservation and enhanced mainstreaming. Notwithstanding much debate on the bold protected area target [24–27], the Post-2020 GBF should still be expected to aim higher on the area-based protection/retention target and regard it as a critical milestone for global biodiversity conservation in the coming decades. Land-use change acted as the sharpest contradictions between nature and man over the past 50 years [4]. The decline of nature will not stop unless biodiversity per se has sufficient space to sustain whilst ensuring man's needs are met guided by the Agenda of Sustainable Development. Bold, as well as deliberate, area-based target will drive transition of contradictions to managed trade-offs between needs of nature and man, by adopting other effective conservation measures (OECMs), spatial planning, and adaptive management and governance interventions. Meanwhile, the use and management of biodiversity from ecosystems to genes to meet humans’ needs must be bounded within the limit of planetary boundaries and fixed on the road of sustainability. Moving forward, we suggest a series of milestones/overarching targets to delineate the step path of reaching the UNM Vision as *‘by 2030, safeguard 1/4 of the Earth with integral, functioning, and connected ecosystems to support* *sustainability both of nature and its contributions to humanity while addressing other direct drivers on biodiversity loss, and step along the path to increase the proportion of the safeguarded Earth to 1/3 by 2040, and 1/2 by 2050 to finally achieve the 2050 Vision – the Unity of Nature and Man’* (Fig. 2).
